# The Use of Saliva as a Biosample in the Light of COVID-19

**DOI:** 10.3390/diagnostics11101769

**Published:** 2021-09-26

**Authors:** Irena Duś-Ilnicka, Elżbieta Krala, Paulina Cholewińska, Małgorzata Radwan-Oczko

**Affiliations:** 1 Oral Pathology Department, Faculty of Dentistry, Wroclaw Medical University, ul. Krakowska 26, 50-425 Wroclaw, Poland; elise7@interia.pl (E.K.); malgorzata.radwan-oczko@umed.wroc.pl (M.R.-O.); 2 Institute of Animal Breeding, Faculty of Biology and Animal Breeding, Wroclaw University of Enviromental and Life Sciences, ul. Chełmońskiego 38C, 51-630 Wroclaw, Poland; paulina.cholewinska@upwr.edu.pl

**Keywords:** salivaomics, biobanking, oral microbiome, SARS-CoV 2, COVID-19

## Abstract

Saliva is easy to collect and a biofluid that is readily available without the need for special equipment for its collection. The collection process, which is non-invasive and inexpensive, leads to obtaining a biomaterial that can serve as a source of information for molecular diagnostics of diseases in general medicine, genetics and dentistry. Unfortunately, many of the salivary methodologies are lacking important parameters to provide for not only the safety of the operator, but also the quality and reproducibility of the research. Since the COVID-19 pandemic, salivary diagnostics demonstrate a great potential for research of SARS-CoV 2. In this review, good practice for unstimulated saliva collection and patient preparation was provided, based on the latest literature and available guidelines. Schemes for saliva collection procedures were presented following an extended literature search. Descriptions of salivary probes/cups, techniques of saliva collection, and the use of specific buffering solutions for the stability of collected samples for SARS-CoV-2 detection were also evaluated.

## 1. Introduction

As a basic biological fluid maintaining homeostasis in the oral cavity, saliva has multiple functions, including tissue lubrication, supporting chewing, eating, swallowing, and digestion processes. Because of its constant flow, saliva provides protection for oral mucosa and the surface of the teeth against mechanical, biological, and chemical factors, i.e., dilution and the ease of swallowing process of acids that might trigger enamel erosion [1,2,3]. This fluid not only plays the role of a crucial factor facilitating both oral and overall health, but also aids in preventing oral diseases. This basic diagnostic biomaterial links different fields of medicine with dentistry and makes a link between patient’ plasma [4,5].

After the outbreak of the SARS-CoV-2 pandemic, saliva has emerged as a convenient and cost-effective point-of-care biomaterial for this recent and most problematic infection’s diagnostics [6,7]. The use of saliva instead of nasopharyngeal swabs is already a common practice, however is not yet standard. A home-based collection of saliva using probes shipped to patients would minimize the direct contact of healthcare workers with potentially infected patients [8]. Additionally, if the correct methodology and concept were developed, the diagnostics of a salivary biomarker correlated with the SARS-CoV-2 virus could be of great importance for the medical field.

Since saliva presents more diagnostic possibilities during the COVID-19 outbreak, the aim of this this semi-systematic review was to summarize the crucial knowledge about salivary excretion, to provide available methods for unstimulated and stimulated saliva collection, to present the possibility of its biobanking, and to discuss the preparation of the patient for different saliva collection processes.

## 2. Materials and Methods

Different search tools were used for the purposes of the non-systematic and systematic parts of the review to ensure the best outcome of the writing process. Chapters without the materials and methods description were based on narrative non-systematic review, and the authors’ laboratory experience. 

For Section 3.2.2 entitled “Pre-analytical laboratory procedures for saliva diagnostic protocol” of the present article, a search of Pubmed.gov (accessed on 9 August 2021) based on the keywords—“saliva AND collection AND storage” was performed. It was narrowed to publications concerning human sampling. Abstracts of articles were evaluated and included in the manuscript when actually containing the valuable requested parameters. Reviews and book chapters were rejected from the search, and only original works were evaluated. 

For Section 3.3 “Salivary diagnostics of SARS-CoV-2” of the present article, a methodology of saliva preparation as an equivalent material for SARS-CoV-2 testing was analysed. For this purpose, a search on Pubmed.gov (accessed on 9 August 2021) based on keywords “saliva AND diagnostic AND SARS-CoV” was performed, and its status was evaluated on 30 August 2020. A total of 83 publications were available as a result of the search. Narrative and systematic reviews along with comments to articles, short communications, and questionnaire-based studies were excluded from the list of publications. Duplicate publications were excluded. After title search and primary evaluation of publications, 39 articles were pre-selected. Finally, 12 publications were included in the research, and these are presented in the table, which includes references that are listed in the reference list. 

## 3. Discussion

### 3.1. Saliva Excretion and Physiology of Salivary Glands

The characteristics of saliva constituents include a disproportionately high volume of salivary flow in relation to the mass of gland tissue. The glandular secretory contribution in this process depends on the type of stimulation. For unstimulated saliva, the submandibular gland is responsible for 65% of salivary excretion, the parotid gland for about 20%, the sublingual gland for about 5%, and minor glands for about 10%. The process of stimulation changes the means of saliva secretion, whereby the parotid gland produces over 50% of all saliva and the submandibular gland about 35%. Additionally, stimulated saliva is produced by minor glands which account for up to 8% each [9,10,11]. Each salivary gland secretes a characteristic type of saliva, with different ionic and protein composition which is of great importance in choice of diagnostics tools. The parotid gland is composed of serous acini and produces saliva of watery and serous structure. The submandibular gland, which plays a major role in unstimulated and stimulated saliva production, has a majority of serous acinar cells with few mucous cells. Also, the sublingual gland is defined as a mixed gland, and contains mucous and serous acini; however, it is composed mostly of mucous acinar cells [9,10,12]. The anatomical architecture of major salivary glands is mostly the same: ductal epithelial cells with secretory endpieces opening into the oral cavity, and the acini which produces saliva. In the ducts, saliva is transported and modified before excretion into the oral cavity [12]. The collected whole saliva apart from glandular secretions contains gingival sulcus fluid, oral microbiome and its metabolites, plaque, epithelial and blood cells, food debris, and various exogenous substances [10]. The secretion and composition of saliva is regulated by both the parasympathetic and sympathetic branches of the autonomic nervous system. The stimulation from the site of the parasympathetic system results in a high flow of watery, serous saliva containing low levels of organic and inorganic components, whereas sympathetic stimulation results in a low volume of saliva with high protein concentration [12]. Also, neurotransmitters, acetylcholine activating muscarinic receptors, and noradrenaline activating adrenergic receptors are involved in salivary gland innervation. Many other neurotransmitters are discussed in the available literature, such as vasoactive intestinal peptide, neuropeptide Y, encephalin, substance P, and neurokinin A as those that play roles in the salivary gland function as well [12]. The specific knowledge about the process of saliva excretion defines salivary constituents, and is crucial to a correct understanding of proper pre-analytical and diagnostic schemes.

### 3.2. Saliva Collection

#### 3.2.1. Procedures including Patient

To eliminate errors, a standardisation of saliva sampling in terms of collection method accuracy, time of day, and other conditions depending on the type of the biomarker to be evaluated is crucial [13]. The preparation for the experiment should begin with an evaluation of the already existing saliva collection protocols and available on the market commercial kits, including an understanding of their limitations. The stimulation of saliva by means of available on the market chemical or mechanical devices was introduced to induce the sympathetic and parasympathetic nerves in order for the salivary gland to start the production of saliva with changed composition [9]. Table 1 presents available commercial and non-commercial collection kits available on the market for the unstimulated and stimulated saliva collection.

Apart from the differences in saliva composition on account of the stimulation process, the differences appear also in unstimulated saliva secretion in terms of the patient’s gender. It was found that higher secretion levels are present in healthy men in comparison to healthy women. A suggestion was made that the cause of this fact could be the difference in the size of salivary glands in males and females, and a gender-dependent saliva secretion process was indicated [14]. Given the above, a proper formation of the patient study group is of great importance. According to WHO Protocol 2010, saliva samples ought to be collected in the morning (9.00 a.m.–11.00 a.m.), on an empty stomach, without drinking any fluids except water, 5 min. after rinsing the mouth thoroughly (with water) and spitting the water without drinking it. The patients/volunteers should spit whole saliva in resting conditions, without talking, with their head down, to make the secretion run naturally during the collection process. Coughing up mucus is not required. Each 60 s, for 10 min, the patients/volunteers should spit saliva into the collection tube. To ensure genuine results, the pH of the saliva should be measured immediately after collection [13]. This protocol presents an accurate method for unstimulated saliva. There is, however, a discrepancy between research done with stimulated saliva and sampling following physical exercises, and with cases when the collected saliva has to present specific contents like hormones [15,16,17]. There are strong correlations between the patient’s preparations for the experiment and no food/oral hygiene before the sampling that are not so widely discussed in the published research. Some scientists took significant effort to put forward specific guidelines to help patients prepare for saliva collection. Howland et al. presented guidelines for female patients for the saliva collection that included avoiding caffeine-based liquids 6 h before sampling, and rinsing the mouth just before sampling as provided in the WHO protocol. Since the research was carried out for the purpose of oxytocin detection by the HPLC method, this standardisation could be of much help [17]. Another example of a strict saliva collection protocol to evaluate levels of salivary biomarkers was provided by Justino et al. [11]. In this experiment, patients were asked to refrain from eating 2 h and drinking water 30 min. prior to saliva collection. The saliva collection was performed between 08:00 a.m. and 09:00 a.m., and five minutes before saliva collection, patients were asked to rinse their mouth with water, which is also referred in the WHO protocol [11]. With regards to saliva composition after tooth brushing with a fluoridated toothpaste, some researchers suggest that the total antioxidant capacity did not change after the oral hygiene procedures, but reduced secretion rates of total protein was presented and increased concentration of alpha-amylases was observed [11].

#### 3.2.2. Pre-Analytical Laboratory Procedures for Saliva Diagnostic Protocol

Pre-analytical parameters should be discussed [14] so that the process of the saliva collection does not interfere with correct diagnostics. Due to the differences in the processing of saliva samples and the temperature of its storage, Table 2 was prepared to compare parameters applied by scientists in recent research protocols.

As shown in Table 2, many research groups prepare strict protocols for saliva processing, including its storage before the experiment and after the sampling on wet ice [18], in 4 °C temperature [15] or with the use of dry ice [17], or the use of stabilisation buffers for the saliva conservation before diagnostics [16,19,20,21], or the application of commercial kits for saliva sampling [20,22,23]. Sharing laboratory and clinical experience for the purposes of patient preparation might help avoiding pre-analytical errors during future experiments of a similar nature. Based on the research carried out for the purposes of this review, we propose the introduction of best practices concerning the standardisation of saliva collection, which are indicated in Table 3. The centrifugation rate shown in this table for lowering the debris presented in saliva, which might influence the laboratory results, was selected to be equal to the centrifugation rate for the patients’ plasma.

#### 3.2.3. Saliva Stability for Diagnostics

Regarding the storage of the salivary samples after collection, the manuals presented by researchers about the proper temperature and time of storage vary, and depend mostly on the research aim. Some researchers suggest that saliva should be stored at room temperature only up to 30–90 min [31]. If the procedure of freezing is not available, samples then should be stored in the temperature of 4 °C only up to 6 h to prevent the degradation of molecules in saliva and to avoid bacterial growth [9]. Thomadaki K et al. claimed, however, that when the salivary proteome is evaluated, the lower the temperature—the better the results [32]. Additionally, it was confirmed that the saliva collection process affects C-reactive protein, and the whole protein concentration [14]. Some laboratory scientists propose, however, that if the experiment is not going to be performed directly after the collection and transport of the samples, freezing at −80 °C or at a temperature below −20 °C is recommended after the pre-analytical procedures in order to avoid possible degradation of stored molecules [15,31]. That storage temperature of −80 °C is often used by biobanks that preserve saliva for pharmaceutical or clinical purposes [15]. In the case of long-term storage, if the saliva sample is intended for high capacity research with the use of NGS platforms for DNA and RNA research or PCR diagnostics, the additional use of stabilisation buffers with specifically designed composition need to be considered [19,20].

#### 3.2.4. Saliva Biobanking

Biobanking is the part of the medical laboratory field research that has systematised the pre-analytical phase and storage of biosamples. The International Agency for Research on Cancer (IARC), the International Society for Biological and Environmental Repositories (ISBER), and the Biobank and Cohort Building Network (BCNet), among others, are the international sources of information and good biobanking practices for different biosamples [33]. Biobanks and biorepositories that adopted best practices could provide crucial information about the samples collected, for example, before the epidemic/pandemic to facilitate the search for the spread of a specific pathogen. This would make it possible to diagnose patients’ samples from specific geographical areas from the time before the epidemics/pandemics, in order to evaluate if the newly discovered microorganisms were affecting the patients before, or to diagnose patients’ old/aged samples with new techniques [34].

Saliva might be self-collected by a volunteer and sent over from a distant location, which makes it possible to conduct longitudinal population-based studies [35,36]. From the technical point of view, saliva needs to be safely stored for it to provide good biobanking material [15,36,37,38]. The patient willingness to participate in the biobanking of body fluids varies internationally [37]. One of the reasons to provide good laboratory practice for its preparation for biobanking is, as reported by Lang et al., that the patient willingness to donate saliva is greater than to donate other biofluids [37]. It is therefore crucial to educate patients about the various open-consent forms for biobanking purposes, in comparison to standard consent forms for research purposes [36,37], and to inform the patient to follow all standard, operational procedures determined for the trial, as all changes in the protocol that are not reported might result in yielding misleading results in the future.

### 3.3. Salivary Diagnostics of SARS-CoV-2

Because of its properties, saliva is considered to be a carrier for oral microbiological composition. The detection of viruses in saliva is mostly based on its nucleic acid evaluation, either by DNA or RNA depending on the type of virus that is evaluated, or in some cases on cell culture [8,39]. With regard to previously described collection methods, fortunately, viral nucleic acid-based methods are considered safer for the operator, because pathogens in the samples might be inactivated with the use of specific inhibition buffers on the initial viral nucleic acid isolation protocol [40].

Due to the spread of the novel coronavirus around the world, the specific, comparable diagnostic methods for detecting SARS-CoV-2 had to be established. After the initial, global struggle to prepare equipment for the mass level of diagnostics required, the procedure was finally established. To this point, nasopharyngeal swabs are still the gold standard in terms of recommended diagnostic biomaterials for the cases of suspected SARS-CoV-2 infection [8]. However, the collection of those types of swabs is not suitable for all types of patients. As reported recently, this process may cause discomfort and bleeding, especially in patients with thrombocytopenia [8], thereby additionally inducing coughing and sneezing in patients [41] and generating aerosols that are potential health hazards for collection personnel. Additionally, saliva can be collected at home, reducing healthcare workers interactions with patients, and therefore also reducing the risk of infection [42]. According to To et al., who analysed posterior oropharyngeal saliva samples of patients with COVID-19 in two hospitals in Hong Kong, the salivary viral load was peaking in the first seven days after symptoms developed and then declined over time [41]. According to the Food and Drug Administration’s Emergency Use Authorization for an assay intended for use in SARS-CoV-2 diagnostics, saliva specimens must be collected, transported, and stored using a specific saliva collection device. During the time of transport ambient temperature can be applied if the testing is performed within 48 h from the collection (see Table 4) [43].

Salivary diagnostics for the purposes of SARS-CoV-2 infection diagnostics might, in the near future, include a handful of methods for patient diagnostics in dental clinics. However, to make these methods work, a proper preparation of dental personnel in the scope of this biomaterial collection is needed. The following crucial parameters seem to be of greatest concern: the proper volume and characteristics (type) of saliva, patients’ preparation for saliva collection, the characteristics of the containers’/used, and the use of stabilisation buffers. Those parameters were summarized in Table 4.

The total volume of saliva collected varied from 1 mL [51], 2 mL [48,54], 3 mL [53], to 5 mL in presented manuscripts [50]. The majority of containers for this biospecimen, especially for patients who were asked to produce posterior oropharynx saliva [49], were sterile urine containers that are not ideal for this purpose, because they are of a larger volume than is required for to saliva capture [50,53]. This type of container, however, has a wider opening, and as such provides an advantage when treating patients who are required to provide the saliva by spitting. There are several viral inhibition and transport media for virus containing specimens on the market, but unfortunately their components are not provided as general information. For these reasons and because of the cost-effectiveness of this process, some researchers provide their own inhibition or inhibition/transport media [52], but do not share full information on their composition. The certification of those media would ensure the safety of operators, and probe, decreasing the workload for laboratories. Unfortunately, viral inhibition medium for the purpose of saliva collection only is difficult to obtain in the commercial market of molecular biology reagents. 

## 4. Conclusions

Saliva provides crucial material for molecular diagnostics in dental studies. There is, however, a need for standardisation in the scope of clinical preparation of patients for studies, along with a high quality of pre-analytical procedures provided during the first hours of sample treatment. Special attention should be given by clinicians to stick with the schemes for the collection of saliva developed during the COVID-19 outbreak. Works related to methodology should be detailed and provided in full to help to reproduce the research for future reference.

## Figures and Tables

**Table 1:** The description of stimulated and unstimulated whole saliva commercial and non-commercial collection procedures and kits.

Types of Saliva Collection		Mechanism	Examples of Commercial Kits	Examples of Non-Commercial Kits
Unstimulated	Spitting	Accumulation and continuous spitting of saliva	Omnigene, DNA Genotec^®^	Spitting saliva into the sterile probe, falcon.
	Passive drool	Accumulation of saliva on the floor of the mouth, patient leaning forward	Salivabio’s Passive Drool, (Salimetrics^®^ Europe Ltd., Suffolk, UK)	Saliva flow into the sterile probe, falcon.
	Soaking—children under 6	Soaking saliva with sorbent	Sorbette (Hydrocellulose, Salimetrics^®^ Europe Ltd., Suffolk, UK)	Cotton swab
Stimulated	Mechanical	Chewing	Salivette^®^, Sarstedt	Chewing gum, Paraffin
	Chemical	Rinsing oral cavity with chemical compound stimulating saliva production	Saliva Collection System (Scs)^®^ (Greiner Bio-One Gmgh, Kremsmuenster, Austria)	Drops of lemon juice

**Table 2:** The comparison of saliva preparation and pre-analytical protocols.

Laboratory Parameters	Article Authors and References Number	Diagnostic Technique	Description of Patients’ Preparation before Saliva Collection	Method of Saliva Collection	Commercial Kit for Saliva Collection	Saliva Storage before Experiment	Addition of Stabilisation Buffer	Volume of Sample	Centrifugation Prior Experiment	Sample Biobanking Temp.
Salivary cortisol levels	Anastasova et al., 2017 [16]	ELISA	No.	Unstimulated saliva: passive drool	No	−80 °C	No.	1 mL	Yes	−80 °C
	Vrbanović, Lapić, Rogić, & Alajbeg, 2019 [24]	ELISA	-7 a.m. And 5 p.m., -2 h no food and oral hygiene, -oral cavity rinsing with water before collection.	Unstimulated saliva: spitting (?)	No	−80 °C	No	5 mL (aliquots of 1 mL each)	1000× *g*, 5 min	−80 °C
	Rolfsjord et al., 2017 [25]	Radioimmunoassay	-6:00 a.m., -fasting saliva.	Unstimulated saliva: soaking of hydrocellulose in saliva	Yes	−86 °C	No	150 µL	Yes	−86 °C
Cotinine	Van Waateringe et al., 2017 [23]	Ultra-high-performance liquid-phase chromatography Phy (uhplc)	-non-fasting saliva, -manufacturer recommendations.	Manufacturer recommendations	Yes	Wet ice	-manufacturer recommendations	~2 mL	2500× *g* for 10 min	−80 °C
	Honarmand, Nakhaee, & Moradi, 2018 [26]	ELISA	-9 a.m.-11 a.m. -rinsing oral cavity with water.	Stimulated saliva: chewing flavour-free gum for 60 s.	No	−70 °C	No	-	No	−70 °C
	Binishabaib et al. [27]	ELISA	-morning hours, -fasting saliva.	Unstimulated saliva: passive drool	No	−70 °C	No	-	3000 rpm for 15 min	−70 °C
Oral microbiome	Goode, Cheong, Li, Ray, & Bartlett, 2014 [19]	NGS	-30 min eating and drinking refrain, -oral cavity rinsing with water before collection.	Unstimulated saliva: spitting (?)	No	Rt *	Yes	2.5 mL	No	<3 months: rt >3 months: 4 °C
	Rodríguez-Rabassa et al., 2018 [22]	PCR technique	No	Unstimulated saliva: passive drool	Yes	−20 °C	No	1 mL	No	-
	Pramanik et al., 2012 [15]	Sequencing 16 s rrna genes	-2 p.m. to 4 p.m.	Unstimulated saliva: passive drool	No	24 h in 4 °C	Yes	5 mL	13,000× *g* for 5 min	−80 °C
Salivary mucins (muc7 muc5b)	Szkaradkiewicz-Karpińska et al. [28]	ELISA	-8 a.m. to 10 p.m., -2 h of eating and drinking withdrawal.	Unstimulated saliva: spitting.	No	−80 °C	No	2 mL	3000× *g* for 15 min in 4 °C	−80 °C
	Ligtenberg, Brand, Van Den Keijbus, & Veerman, 2015 [29]	-ELISA, -westernblot, -sds-page gels	-9 a.m. to 11 a.m. -preparation of patients and samples according previous research	Unstimulated saliva: expectoration	No	−20 °C	Yes	-	10,000× *g* for 5 min	-
	Gabryel-Porowska et al., 2014 [30]	ELISA	-9 a.m. to 11 a.m. -no further description of preparation	Unstimulated saliva: spitting	No	−70 °C	No	-	10,000× *g* for 15 min in 4 °C	−70 °C
	Pramanik et al., 2012 [15]	SDS-page gels	-2 p.m. to 4 p.m. -no further description of preparation	Unstimulated saliva: passive drool	No	24 h in 4 °C	Yes	5 mL	No	−80 °C

* Legend: Unstimulated: u/s; room temperature: rt; data not available: N/A; PCR: Polymerase Chain Reaction; CMV: Cytomegalovirus; CK: commercial kit; HPLC: high-performance liquid chromatography; UHPLC: ultra-high-performance liquid-phase chromatography; Next Generation Sequencing: NGS; ?, the data is not clear in the article.

**Table 3:** Good laboratory practice for unstimulated saliva collection and patient preparation, based on latest literature and WHO guidelines.

Remarks	Description of Steps
Patient preparation for saliva collection:	1. Patient does not eat, drink or perform oral hygiene for two hours before saliva collection.
	2. Patient washes mouth with lukewarm water, and spits it without swallowing.
	3. Patient waits 5 min after washing mouth with water.
Whole saliva collection up to 10 min	4. Saliva is collected by spitting, after natural secretion without stimulation, material is collected to sterile 5 mL volume probe.
Probe is placed on ice during transport and pre-analytical preparations	5. pH is measured and result is recorded in patients’ chart.
	6. Saliva is mixed with a sterile disposable pipette to homogenise the probe.
	7. Addition of a stabilisation buffer for the time of transport to the laboratory (step to be evaluated if the method allows it).
Laboratory proceedings for the saliva preparation:	8. Centrifugation in 4 °C, 2500 rpm.
	9. Supernatant is collected, and precipitate is disposed.
	10. Store at −80 °C.

**Table 4:** The comparison of technical and pre-analytical preparation of patients for SARS-CoV-2 salivary diagnostics.

**References**	**Type of Saliva**	**Saliva Collection Point**	**Patient Preparation for Saliva Collection**	**Addition of Viral Transport Medium**	**Time of Saliva Collection**
[41]	Posterior oropharynx saliva	Hospital	No oral hygiene and eating before saliva collection.	Yes	Early morning after awakening
[44]	Whole saliva, by drooling technique	Hospital	ND *	No	ND
[45]	Whole saliva, by drooling technique	Self-collection	No oral hygiene and eating 10 min. before saliva collection. Rinsing mouth with water 5 min before saliva collection.	Yes	ND
[46]	Whole saliva, by spitting technique	Supervised self-collection	No information about baseline probe collection. Chlorhexidine gluconate mouthwash used before next collection	Yes	ND
[47]	Whole saliva, by spitting technique	Supervised self-collection	No oral hygiene and eating 5 min. before saliva collection. Rinsing mouth with water.	Yes	ND
[48]	Posterior oropharynx saliva	Supervised self-collection	No oral hygiene and eating 5 min. before saliva collection. Rinsing mouth with water.	Yes	Early morning
[49]	Posterior oropharynx saliva	Self-collection to sterile urine cap	No oral hygiene, eating, and drinking before saliva collection	No	Early morning after awakening
[50]	Whole saliva	Supervised self-collection to sterile urine cap	No oral hygiene, eating, and drinking, tobacco, or gum for 30 min before saliva collection	No	ND
[51]	Whole saliva, by spitting technique	Supervised self-collection to Spectrum Solutions LLC SDNA-1000 Saliva Collection Device	ND	Yes	ND
[52]	Posterior oropharynx saliva	Supervised self-collection	ND	Viral transport medium prepared at home	ND
[53]	Whole saliva, by spitting technique	Supervised self-collection to sterile urine cap	ND	No viral transport media, nor stabilising agents	ND
[54]	Posterior oropharynx saliva	Supervised self-collection to sterile specimen bottle	1: No oral hygiene, eating, and drinking before saliva collection.2–5: no special requirements.	Yes	1. Early-morning; 2. Before lunch; 3. Before afternoon tea at 3 p.m.; 4. Before dinner; 5. Before bedtime.

* Legend: ND: not disclosed in the research.

## Data Availability

Not applicable.
